# Effect of Al_2_O_3_ with Different Nanostructures on the Insulating Properties of Epoxy-Based Composites

**DOI:** 10.3390/ma13194235

**Published:** 2020-09-23

**Authors:** Yongzhe Tang, Guanghui Ge, Yuxia Li, Liangsong Huang

**Affiliations:** Key Laboratory for Robot Intelligent Technology of Shandong Province, Shandong University of Science and Technology, Qingdao 266590, China; yztang@sdust.edu.cn (Y.T.); ghge@sdust.edu.cn (G.G.); yuxiali2004@sdust.edu.cn (Y.L.)

**Keywords:** nanostructures, thermal conductivity, volume resistivity, dielectric performance, AC breakdown performance

## Abstract

High thermal conductivity insulating dielectrics with good electrical properties have received widespread attention due to the continuous development of power systems and power electronic technologies. In this paper, the effects of differently structured nano alumina fillers on the thermal conductivity and insulating properties of polymer-based composites were studied. It was found that all three types of Al_2_O_3_ nano-fillers enhanced the thermal conductivity of the composites, and the thermal conductivity increased more dramatically with increasing filler particle size. It is worth noting that Al_2_O_3_ nanowires (NWs) exhibited the most significant improvement in thermal conductivity. The volume resistivity of the composites first increased and then decreased with increasing mass fraction of fillers, and Al_2_O_3_ nanoplates (NPLs) showed the most significant improvement in the insulation performance of the composites. The dielectric constants of the composites increased with increasing mass fraction of fillers, while the dielectric losses first decreased and then increased with the same trend, yet the mass fractions of fillers for the three materials were different when the dielectric loss reached a minimum. In addition, all three types of filler increased the AC breakdown strength of the composites, but Al_2_O_3_-NPLs showed the most significant improvement on the breakdown performance of the composites.

## 1. Introduction

Polymers are widely used for dielectric energy storage and high voltage insulation materials due to their better mechanical and electrical properties compared with traditional ceramic materials [1,2]. More specifically, epoxy resins exhibit good dielectric and breakdown properties, and show good fire and stretching resistance, making them one of the best choices in the field of high-voltage insulation [3,4,5,6,7]. Additionally, the integration of the power electronics industry has significantly increased, and dielectric film capacitors are becoming more relevant with the rapid development of power systems. Hence, the requirements for the dielectric and breakdown performance of polymers are becoming stricter [8,9,10,11]. Moreover, the thermal conductivity of polymers is generally low due to the complex morphology of the polymer chains. For example, the thermal conductivity of epoxy resins is approximately 0.20836 W/m·K. The poor heat conduction ability of polymers leads to poor insulation performance and reduced lifespan of power electronic equipment, limiting its application in certain fields, such as polymer-based flexible electronics [12,13,14]. Polymer-based composites have received extensive attention as an alternative to improve the thermal and electrical properties of polymers to meet the increasing high thermal conductivity demand for insulating materials in numerous fields [15,16]. Compared with traditional micro-fillers, epoxy-based composites with nano-fillers have better dielectric and breakdown properties [17]. Among the nano-fillers, nano-Al_2_O_3_ has become a current research focus due to its relatively high thermal conductivity, low cost, and the ability to improve the insulation and dielectric properties of the composites based on trap characteristics. The thermal conductivity of Al_2_O_3_ is approximately 35 W/m·K, which is much higher than that of epoxy resins. Therefore, nano-Al_2_O_3_ fillers can improve the thermal conductivity of composites, which has reached 0.55 W/m·K when the mass fraction of the filler was 80%, increasing by 264% compared with the pure epoxy resin [18,19,20]. Moreover, it has been proved that the thermal conductivity of the composite depends on the particle size of the filler. The addition of Al_2_O_3_ to epoxy resins introduces deep traps, which increase the volume resistivity, improve the insulation performance, and reduce the dielectric loss of the composite to a certain extent [21]. In addition, the high dielectric constant of Al_2_O_3_ increases the dielectric constant of the composite [22,23,24]. With increased volume resistivity and reduced dielectric loss, the electrothermal breakdown performance of the composite under an AC electric field was enhanced [25,26]. Most of the above conclusions were derived from experiments using 0-D Al_2_O_3_ particles. However, the influence of Al_2_O_3_ fillers with different nanostructures including nanoparticles (NPs), nanowires (NWs) and nanoplates (NPLs) on the properties of the composite has not been studied. The nanostructure of the filler is an important factor in improving the performance of the composite, and it has been stated that 1-D and 2-D nanostructured fillers can improve the performance of composites more significantly than their 0-D analogs [27,28]. Therefore, it is of great significance to study the influence of differently structured nano-fillers on the thermal conductivity and electrical properties of polymer-based composites.

In this paper, the effects of the addition of differently nanostructured Al_2_O_3_ fillers on the thermal conductivity and insulating properties of epoxy-based composites were studied. First, a hydrothermal method was used to prepare Al_2_O_3_ fillers with different nanostructures, which were then mixed with an epoxy resin to prepare Al_2_O_3_/epoxy composites with different mass fractions. The structures and nanostructures of the fillers and composites were analyzed by scanning electron microscopy (SEM), and the influence of the differently nanostructured Al_2_O_3_ fillers on the thermal conductivity of the composites was analyzed. In addition, the mechanism by which the different nanostructures of the fillers impacted the electrical properties of the composites, including volume resistivity, dielectric constant, dielectric loss, and AC breakdown strength, were extensively studied.

## 2. Materials and Methods

### 2.1. Materials

Bisphenol epoxy resin (Eponex 1513, E-51), which is later referred to as EP, and anhydride curing agent (methyl tetrahydrophthalic anhydride) were purchased from Changzhou Runxiang Chemical Co., Ltd., Changzhou, China. The accelerator 2,4,6-tris (dimethylamino) phenol (DMP-30) was produced by Shanghai Aladdin Biochemical Technology Co., Ltd., Shanghai, China, and the aluminum sulfate octadecahydrate was produced by Tianjin Damao Chemical Reagent Factory, China. Ammonium hydroxide was purchased from Chengdu Kelong Chemical Co., Ltd., Chengdu, China, and high-purity Al_2_O_3_-NPs were produced by Nanjing Xianfeng Nano Material Technology Co., Ltd., Nanjing, China, with a particle size of approximately 60 nm.

### 2.2. Preparation of h-BN/EP Composites

A hydrothermal method was performed in this experiment. Aluminum sulfate octadecahydrate was magnetically stirred in deionized water, and ammonium hydroxide was added as a precipitant to adjust the pH. The pH values of the mixed solutions were adjusted to 5 and 9 to prepare the precursors of Al_2_O_3_-NWs and Al_2_O_3_-NPLs, respectively. Subsequently, the precursor solution was placed in an autoclave at 180 °C for 10 h to produce aluminum hydroxide powder, which was soaked with deionized water and alcohol, and then dried at 80 °C for 10 h. Finally, the obtained aluminum hydroxide powder was treated at 600 °C for 2 h to obtain Al_2_O_3_-NWs and Al_2_O_3_-NPLs similar to the nanostructures of the precursors.

A blending method was used to prepare epoxy-based composites filled with different mass fractions of Al_2_O_3_-NWs. First, the epoxy resin was heated in an oil bath at 60 °C until it was not adhesive, and the weighed Al_2_O_3_ nano-filler with the corresponding mass fraction was added to the epoxy resin matrix and stirred until uniformly mixed. Subsequently, methyl tetrahydrophthalic anhydride was added to the mixture, dispersed, and emulsified for 30 min. Finally, 2, 4, 6-tris (dimethylamino) phenol accelerator was added and stirred for 30 min. The mixed sample was put into a vacuum box for degassing, poured into a mold, and heated to solidify, and sheet-like samples with a thickness of approximately 1cm were obtained [29].

### 2.3. Performance Tests

#### 2.3.1. SEM Imaging

SEM (Apreo SEM, Thermo Fisher Scientific Co., Ltd., Waltham, MA, USA) was used to observe the nanostructures of the three Al_2_O_3_ nano-fillers and their dispersion conditions in the epoxy resin matrix. The composite materials were cooled in liquid nitrogen and broken to visualize their nanostructures. Then, conductive gold was sprayed on the interfaces of the three different fillers and composite materials.

#### 2.3.2. Thermal Conductivity

A multifunctional thermal conductivity tester (DRE-111, Xiangtan Xiangyi Instrument Co., Ltd., Xiangtan, China) was used to measure the thermal conductivity of the composites in the direction perpendicular to the plane by the Hot Disk method. The sample thickness was approximately 1 mm [30].

#### 2.3.3. Volume Resistivity

A high insulation resistance tester (ZC36, Shanghai Anbiao Electronics Co., Ltd., Shanghai, China) was used to measure the volume resistivity of the composites. A three-electrode connection method was used to connect the shield electrode, the protected electrode, and the measuring electrode. A voltage of 1000 V was applied to both sides of the composites. The average volume resistance within 20 min of the applied voltage was used to calculate the volume resistivity, in order to avoid the interference of the polarization current and environmental factors. The volume resistivity can be obtained as follows:

(1) $$\rho v=Rv\frac{Ae}{t}$$

where ρv is the volume resistivity, Rv is the volume resistance, Ae is the effective area of the protected electrode, and *t* is the thickness of the sample.

#### 2.3.4. Dielectric Performance

A high-frequency LCR digital bridge (TH2826, Changzhou Tonghui Electronics Co., Ltd., Changzhou, China) was used to measure the dielectric loss and capacitance of the composites. The shield electrode, the protected electrode, and the measuring electrode were connected using a three-electrode connection mode before the measurement, with the composites connecting the shield electrode and the protected electrode. The gap of the measuring electrode and the diameter were recorded to calculate the dielectric constant, which can be obtained by Equation (2):

(2) $$\varepsilon_{r}=\frac{4*C*h}{\pi (d_{1}+g{)}^{2}\varepsilon_{0}}$$

where *C* is the capacitance of the sample, *h* is the thickness of the sample, d_1_ is the diameter of the protected electrode, g is the electrode gap, and $\varepsilon_{0}$ is the vacuum dielectric constant.

#### 2.3.5. Breakdown Strength

Computer-controlled AC voltage breakdown test equipment (HJC-100KV, Huabo Technology Industry Co., Ltd., Changchun, China) was used to measure the AC breakdown strength of the composites. The composites were placed between spherical electrodes with a diameter of 25 mm and filled with transformer oil as an insulating medium. A 50 Hz AC voltage was applied between the electrodes, and the voltage between the electrodes was increased at a speed of 1 kV/s. The ratio of the breakdown voltage to the thickness of the composites was used to calculate the AC breakdown strength of the composites.

## 3. Results and Discussion

### 3.1. SEM Imaging

**Figure 1:**
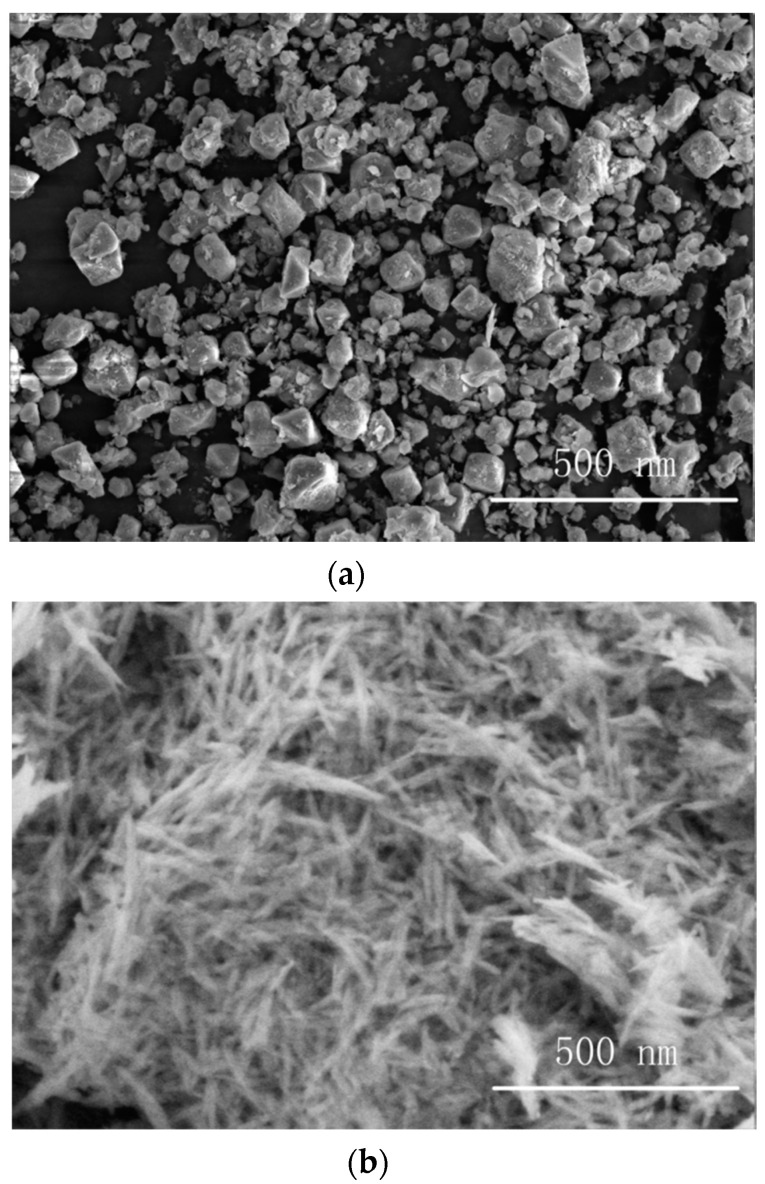

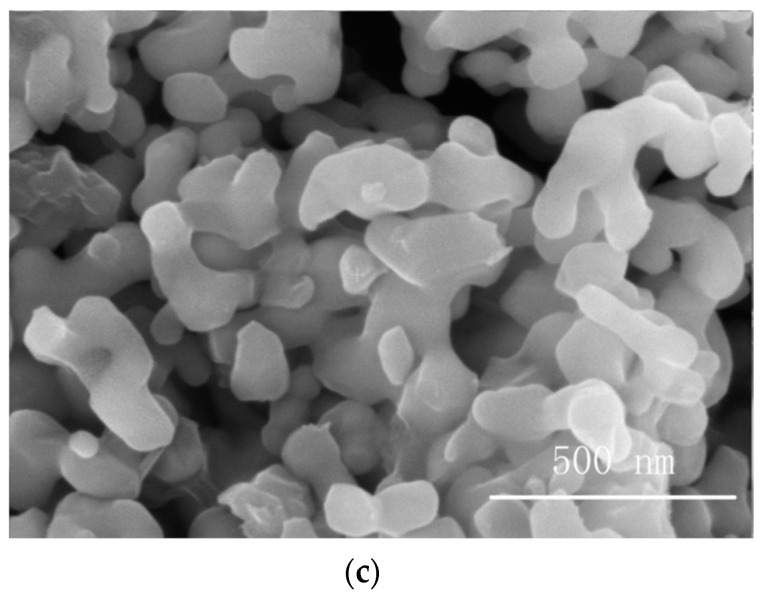
SEM images of (**a**) Al_2_O_3_-NPs, (**b**) Al_2_O_3_-NWs, and (**c**) Al_2_O_3_-NPLs.

Figure 1a shows the SEM image of Al_2_O_3_-NPs. It can be seen that Al_2_O_3_-NPswere in the shape of blocks with sizes of approximately 60 nm, and were well dispersed without obvious agglomeration.

Figure 1b displays the SEM image of Al_2_O_3_-NWs. It can be seen that Al_2_O_3_-NWs were linear with a length of approximately 100 nm and a diameter of approximately 8 nm, uniformly distributed with a disordered orientation and clear interfaces. Moreover, they were well dispersed without obvious agglomeration.

Figure 1c shows the SEM image of Al_2_O_3_-NPLs. It can be seen that Al_2_O_3_-NPLs consisted of clear flakes with a width of approximately 400 nm and a thickness of 50 nm, and were well dispersed. The orientation of the nanoplates was mostly consistent, forming a layered structure, which was different from the first two Al_2_O_3_ fillers.

**Figure 2:**
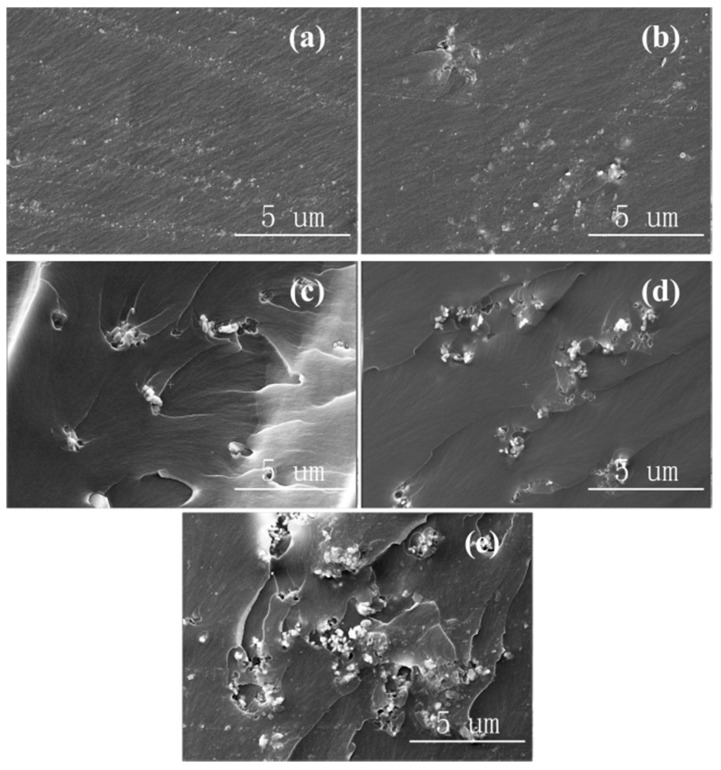
SEM images of Al_2_O_3_-NPs/epoxy resin composites with (**a**) 1 wt.%, (**b**) 3 wt.%, (**c**) 5 wt.%, (**d**) 7 wt.%, and (**e**) 10 wt.% of Al_2_O_3_ filler.

Figure 2 shows the SEM images of Al_2_O_3_-NPs/epoxy resin composites with different mass fractions of Al_2_O_3_-NPs. As shown in Figure 2a, Al_2_O_3_-NPs were uniformly distributed in the epoxy resin matrix without obvious agglomeration at low Al_2_O_3_-NPs mass fractions. However, the composites exhibited a slight agglomeration as the fraction increased to 3% (Figure 2b), and the degree of agglomeration increased as the mass fraction further increased.

**Figure 3:**
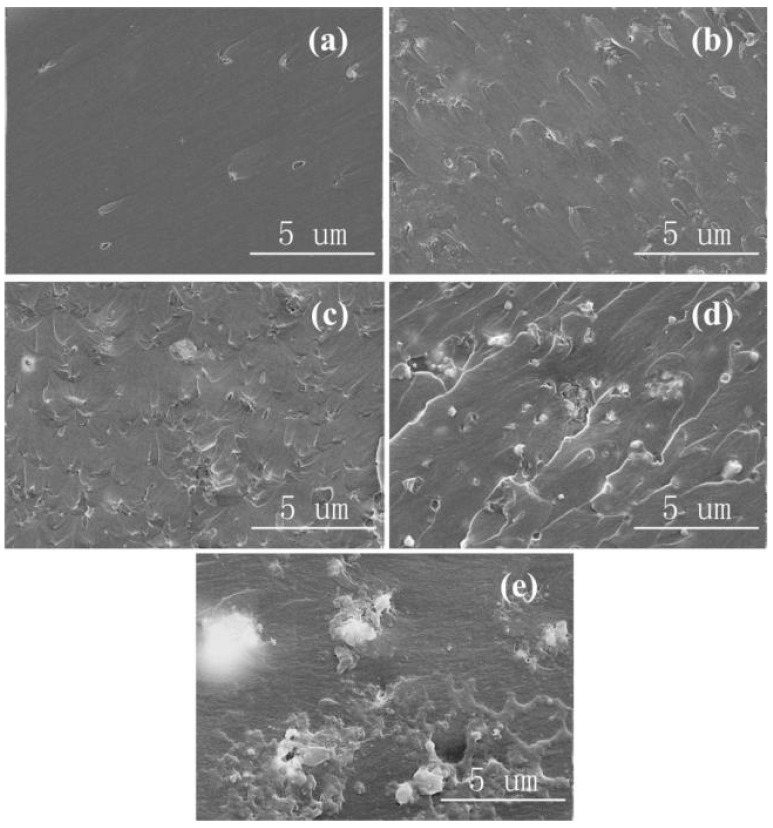
SEM images of Al_2_O_3_-NWs/epoxy resin composites with (**a**) 1 wt.%, (**b**) 3 wt.%, (**c**) 5 wt.%, (**d**) 7 wt.%, and (**e**) 10 wt.% of Al_2_O_3_ filler.

Figure 3 shows the SEM images of Al_2_O_3_-NWs/epoxy resin composites with different mass fractions of Al_2_O_3_-NWs. Similar to the SEM images of the Al_2_O_3_-NPs/epoxy resin composites, Al_2_O_3_-NWs were evenly distributed in the epoxy resin matrix without obvious agglomeration when the mass fraction of filler was 1 wt.%, as shown in Figure 3a. In addition, the Al_2_O_3_-NWs/epoxy resin composite was still well dispersed without agglomeration when the fraction reached 3 wt.% due to the large particle size of the Al_2_O_3_-NWs (Figure 3b). However, after the mass fraction of filler reached 5 wt.%, the composite showed a slight agglomeration and it became more significant with increasing mass fraction of the filler, as shown in Figure 3c–e.

**Figure 4:**
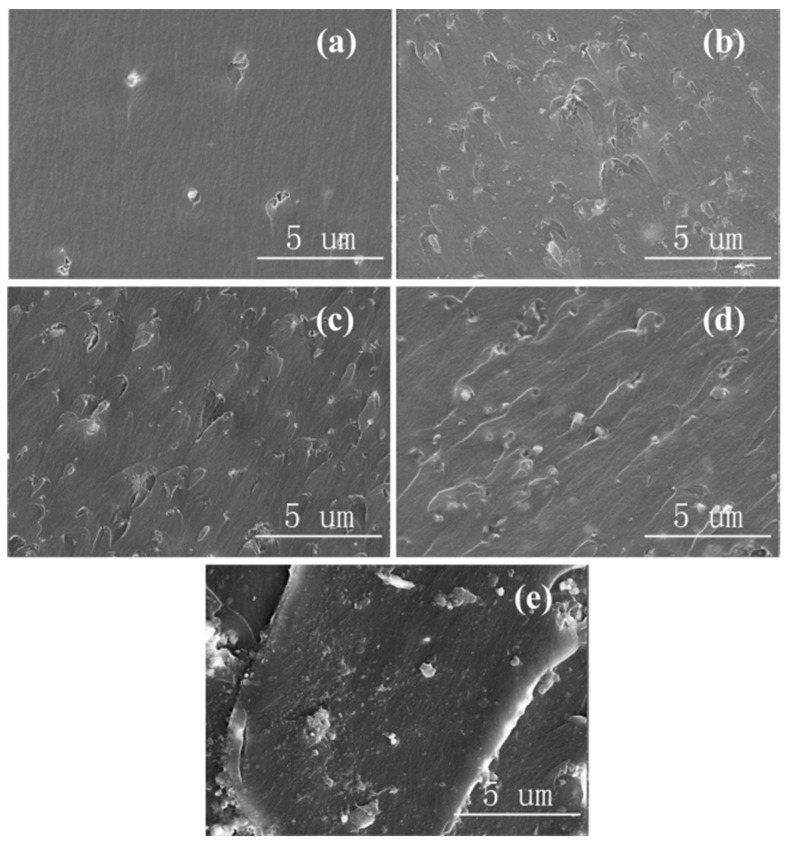
SEM images of Al_2_O_3_-NPLs/epoxy resin composites with (**a**) 1 wt.%, (**b**) 3 wt.%, (**c**) 5 wt.%, (**d**) 7 wt.%, and (**e**) 10 wt.% of Al_2_O_3_ filler.

Figure 4 shows the SEM images of Al_2_O_3_-NPLs/epoxy composites with different mass fractions of Al_2_O_3_-NPLs. The SEM images were somewhat similar to those of Al_2_O_3_-NWs, since their particle sizes were similar, as shown in Figure 4a,b. Al_2_O_3_-NPLs were uniformly distributed in the epoxy resin matrix at low mass fractions of filler (1 wt.% and 3 wt.%). Moreover, Al_2_O_3_-NPLs were still well dispersed without obvious agglomeration when the mass fraction of the Al_2_O_3_-NPLs reached 5 wt.%, as shown in Figure 4c. This can be attributed to the difference in aspect ratio and particle size between Al_2_O_3_-NPLs and Al_2_O_3_-NWs. The obvious agglomeration can only be observed when the mass fraction of Al_2_O_3_-NPLs reached 7 wt.%, as shown in Figure 4d,e.

### 3.2. Thermal Conductivity

**Figure 5:**
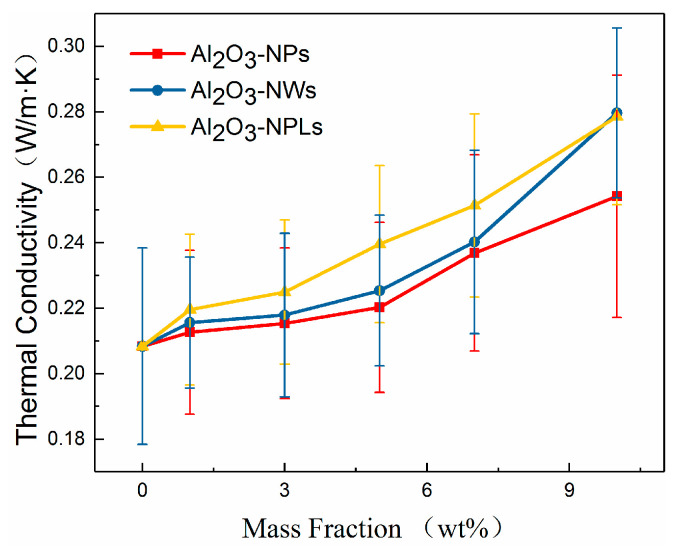
Relationship between the thermal conductivity of composites with the mass fraction of Al_2_O_3_ nano-fillers.

Figure 5 shows the thermal conductivity of epoxy-based composites with differently nanostructured Al_2_O_3_ fillers. It was observed that the thermal conductivity of the composites with Al_2_O_3_ fillers increased when the mass fraction of filler increased and was higher than that of the pure epoxy resin. This can be attributed to the higher thermal conductivity of the Al_2_O_3_ fillers compared with the epoxy resin, and low thermal resistance of the interfacial area formed between the nanostructured Al_2_O_3_ fillers and the epoxy resin. The space between Al_2_O_3_ fillers and the distribution density of the interfacial zone decreased with the increasing mass fraction of the filler, which promoted the formation of a heat-conducting path with low thermal resistance and hence increased the thermal conductivity of the composite.

The differently nanostructured Al_2_O_3_ fillers distinctively enhanced the thermal conductivity of the composites. For example, Al_2_O_3_-NPs cannot form an effective heat-conducting path after being uniformly mixed in the epoxy resin matrix due to their low aspect ratio and small particle size. As can be seen in Figure 5, the thermal conductivity of the Al_2_O_3_-NPs/epoxy composite was significantly lower than that of the other two samples. In contrast, the interfaces formed between Al_2_O_3_-NWs and Al_2_O_3_-NPLs, and the epoxy resin matrix was easily connected, thus forming a heat-conducting path with low thermal resistance, due to their high aspect ratios. In addition, Al_2_O_3_-NPLs had a larger particle size, exhibiting greater thermal conductivity enhancement at low mass fractions of filler. It is worth noting that the improvement in the thermal conductivity of the epoxy resin composite with Al_2_O_3_-NWs was greater than that of Al_2_O_3_-NPLs when the mass fraction of the filler reached 10 wt.%. It can be seen from the SEM images that most of the Al_2_O_3_-NPLs exhibited the same orientation and displayed a layered structure, roughly maintaining their original orientation in the composite after being dispersed in the epoxy resin matrix. This is likely due to the low viscosity of the composite at low mass fractions of filler. Although the enhancement of the thermal conductivity of composites with Al_2_O_3_-NPLs was larger than that of composites filled with Al_2_O_3_-NPs due to their larger aspect ratios, the thermal resistance between layers was hardly reduced. On the contrary, Al_2_O_3_-NWs were more randomly distributed, making it difficult for the fillers to connect and form heat-conducting paths. This is easier when the fillers are evenly distributed in the epoxy resin matrix, thus the thermal conducting performance of the composites is more significantly improved.

### 3.3. Volume Resistivity

**Figure 6:**
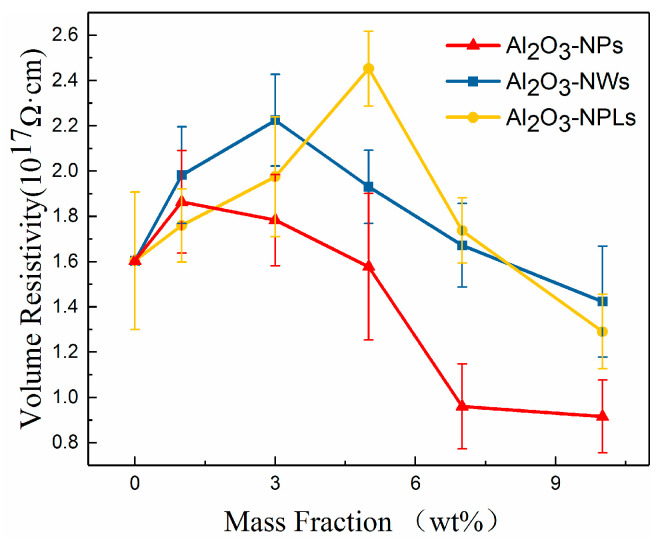
Relationship between the volume resistivity of the composites with the mass fraction of Al_2_O_3_ nano-fillers.

Figure 6 shows the volume resistivity of the epoxy-based composites with different nanostructured Al_2_O_3_ fillers.

It can be seen that the volume resistivity of the three samples first increased and then decreased with increasing mass fraction of filler, indicating that an appropriate proportion of nano-Al_2_O_3_ filler can enhance the insulation performance and reduce the electrical conductivity of the composite.

When an appropriate amount of nano-Al_2_O_3_ filler was added to the epoxy resin matrix, nano-Al_2_O_3_ was tightly combined with the epoxy resin segment to form an interfacial area. Inside this area, the movement of the polymer segment was impeded and its conformation was changed, introducing deep traps within the composite. These deep traps trapped carriers and produced hopping conductivity, which reduced the mobility of the carriers, and thus improved the volume resistivity of the composite. However, as the mass fraction of nano-Al_2_O_3_ fillers increased, the volume resistivity of the composite did not increase further. According to the electric double layer model proposed by Lewis, the interfacial area formed between the filler and the epoxy resin matrix consisted of a Gouy–Chapman diffusion double layer with the same thickness. When the space between Al_2_O_3_ particles (D) was less than twice the thickness of the interface, the interfacial zones began to overlap, the energy level and density of deep traps decreased, and the volume resistivity of the composite decreased [31,32,33].

When nano-Al_2_O_3_ particles are uniformly distributed in the epoxy resin matrix, *D* can be expressed as follows [34]:

(3) $$D=\langle {\left\{\frac{\pi }{6}\frac{\rho_{1}}{\rho_{2}}\frac{100}{\mathrm{wt}\%}\left[1-\frac{\mathrm{wt}\%}{100}(1-\frac{\rho_{2}}{\rho_{1}})\right]\right\}}^{\frac{1}{3}}-1\rangle d$$

where $\rho_{1}$ and $\rho_{2}$ are the density of the Al_2_O_3_ and the epoxy resin, respectively, wt.% is the mass fraction of the fillers, and d refers to the particle size of the Al_2_O_3_-NPs.

According to Equation (3), *D* is inversely proportional to the mass fraction of the filler and directly proportional to its particle size. The volume resistivity of the composite decreases with increasing mass fraction of filler when *D* is greater than twice the thickness of the interface, yet increases with increasing mass fraction of filler when *D* is less than twice the thickness of the interface. It can be seen from Figure 6 that the volume resistivity of the three composites with nano-Al_2_O_3_ fillers first increased and then decreased with increasing mass fraction of filler, which is in good agreement with the analysis of Equation (3), indicating that an appropriate nano-Al_2_O_3_ filler composition can effectively increase the volume resistivity of the composite. In addition, due to the difference in particle size of nanoparticles, nanowires and nanoplates, the distance between the particles of the three alumina fillers with the same mass fraction also increased in turn, and the mass fraction of the fillers with the maximum volume resistivity obtained by the three composite materials also increased in turn. As shown in Figure 6, the maximum volume resistivity of the composite corresponded to 1% Al_2_O_3_-NPs, 3% Al_2_O_3_-NWs, and 5% Al_2_O_3_-NPLs, which is consistent with the above-mentioned analysis. It is worth noting that the maximum volume resistivity of Al_2_O_3_-NPs/epoxy resin, Al_2_O_3_-NWs/epoxy resin, and Al_2_O_3_-NPLs/epoxy resin increased sequentially, indicating that Al_2_O_3_-NPLs exhibited the maximum enhancement of the volume resistivity of the composites. This is likely because Al_2_O_3_-NPLs formed layered structures inside the composites more easily, the space of the particles was larger than the calculated value, and the energy level and density of the deep traps were higher compared with the other fillers, all leading to a higher resistivity in the vertical direction.

### 3.4. Dielectric Performance

In order to describe the influence of the differently nanostructured fillers on the dielectric constant of the composites, the Jayasunderd–Smith (JS) model was used to describe the change in dielectric constant with the mass fraction of filler for Al_2_O_3_-NPs/epoxy resin composites. However, this model cannot be used to describe the relationship between the dielectric constant of the composites and the mass fraction of Al_2_O_3_-NWs and Al_2_O_3_-NPLs due to their different shapes and directions. Hence, the Polder-Van Santen (PVS) model was used for analyzing the dielectric constant of composites with these two fillers. 

The JS model [35] can be described as follows:

(4) $$\varepsilon_{c}=\frac{f_{p}\varepsilon_{p}+f_{f}\varepsilon_{f}[\frac{3\varepsilon_{p}}{\varepsilon_{f}+2\varepsilon_{p}}][1+\frac{3f_{f}(\varepsilon_{f}-\varepsilon_{p})}{\varepsilon_{f}+2\varepsilon_{p}}]}{f_{p}+f_{f}[\frac{3\varepsilon_{p}}{\varepsilon_{f}+2\varepsilon_{p}}][1+\frac{3f_{f}(\varepsilon_{f}-\varepsilon_{p})}{\varepsilon_{f}+2\varepsilon_{p}}]}$$

where $\varepsilon_{p}$ and $f_{p}$ are the dielectric constant and mass fraction of the epoxy resin matrix, respectively, and $\varepsilon_{f}$ and $f_{f}$ are the dielectric constant and fraction of the Al_2_O_3_-NPs, respectively.

The PVS model [36] can be described as follows:

(5) $$\varepsilon_{eff}=\varepsilon_{b}+\frac{\frac{\rho_{b}f_{a}}{\rho_{a}f_{b}+\rho_{b}f_{a}}(\varepsilon_{a}-\varepsilon_{b})}{3}\sum {}_{v=x,y,z}\frac{\varepsilon_{eff}}{\varepsilon_{eff}+N_{v}(\varepsilon_{a}-\varepsilon_{eff})}$$

where $\varepsilon_{a}$, $f_{a}$ and $\rho_{a}$ are the dielectric constant, mass fraction, and density of Al_2_O_3_ nano-fillers, respectively, and $\varepsilon_{b}$, $f_{b}$ and $\rho_{b}$ are the dielectric constant, mass fraction, and density of the epoxy resin matrix, respectively. $N_{v}$ is the depolarization factor, which is a constant describing the degree of depolarization of the nanofiller, related to its shape and direction, and can be expressed as

(6) $$N_{v}=\frac{a_{x}a_{y}a_{z}}{2}\int_{0}^{\infty }\frac{1}{(s+a_{x}{}^{2})\sqrt{\left(s+a_{x}{}^{2})(s+a_{y}{}^{2})(s+a_{z}{}^{2}\right)}}ds$$

where $a_{x}$, $a_{y}$ and $a_{z}$ are the semiaxes of the ellipsoid, namely, the depolarization factors. The depolarization factors $a_{x}$, $a_{y}$ and $a_{z}$ of Al_2_O_3_-NWsare 0, 1/2, and 1/2, respectively. The depolarization factors of Al_2_O_3_-NPLs are 1, 0, and 0, respectively. 

**Figure 7:**
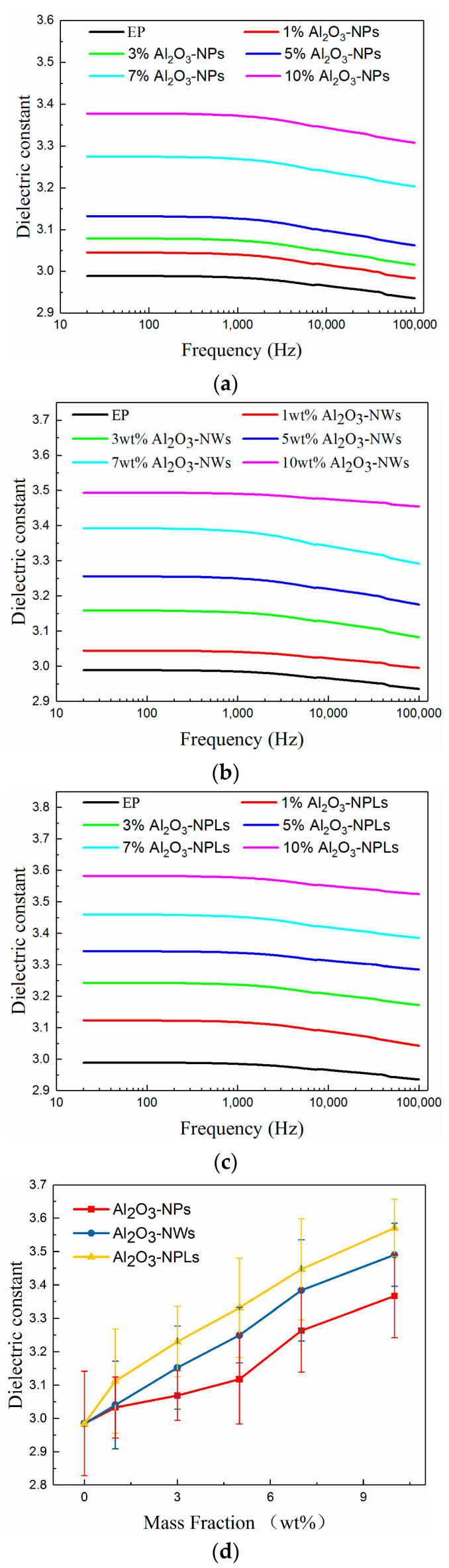
Change in the dielectric constants of the composites of different mass fractions of Al_2_O_3_ nano-fillers with frequency: (**a**) Al_2_O_3_-NPs, (**b**) Al_2_O_3_-NWs, (**c**) Al_2_O_3_-NPLs, and (**d**) dielectric constants of the composites as a function of filler content measured at room temperature and 1 kHz.

It can be seen from the equations that the dielectric constant of the composite increases with increasing mass fraction of filler for both models, and it can be seen from Figure 7 that the experimental results are in good agreement with this analysis. It is worth noting both the theoretical and experimental results show that the effect of Al_2_O_3_-NWs and Al_2_O_3_-NPLs in increasing the dielectric constants of the composites is more pronounced than the effect of Al_2_O_3_-NPs. This may be due to the fact that Al_2_O_3_-NWs and Al_2_O_3_-NPLs have larger aspect ratios than Al_2_O_3_-NPs, resulting in larger dipole moments.

Moreover, the simple static dielectric constant can be expressed as [37]:

(7) $$\varepsilon =1+\frac{\langle M^{2}\rangle -{\langle M\rangle }^{2}}{3\varepsilon_{0}vk_{b}T}$$

where *M* is the vector sum of all dipole vectors, $\varepsilon_{0}$ is the vacuum permittivity, v is the volume of the system, $k_{b}$ is the Boltzmann-constant, and *T* is the temperature. Equation (7) suggests that the static dielectric constants obtained for Al_2_O_3_-NWs and Al_2_O_3_-NPLs are significantly greater than that of Al_2_O_3_-NPs due to their larger dipole vectors, which is consistent with the theoretical and experimental results as well.

It can be seen from Figure 7 that the dielectric constants of the composites remain unchanged with increasing frequency in the low-frequency region. However, the change in the dipole vector was slower than the change in the applied electric field when the frequency further increased, and the polarization performance of the composites weakened. Therefore, the dielectric constant first increased and then decreased with increasing frequency in the high-frequency region.

This is because the electric dipole vector inside the composites will generate an inductive electric dipole vector in the opposite direction to the electric field, and the polarization effect will occur. The electric dipole vector can be turned in the electric field change period at low frequencies while increasing frequency does not significantly affect the dielectric constant of the composite. 

**Figure 8:**
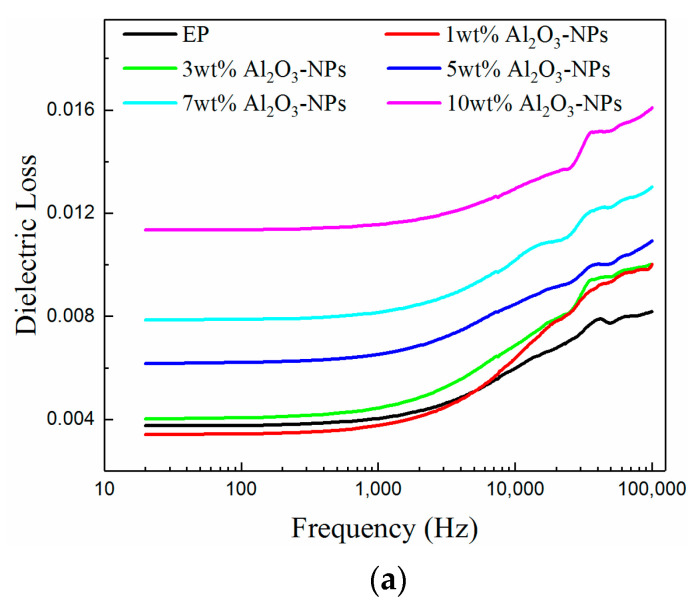

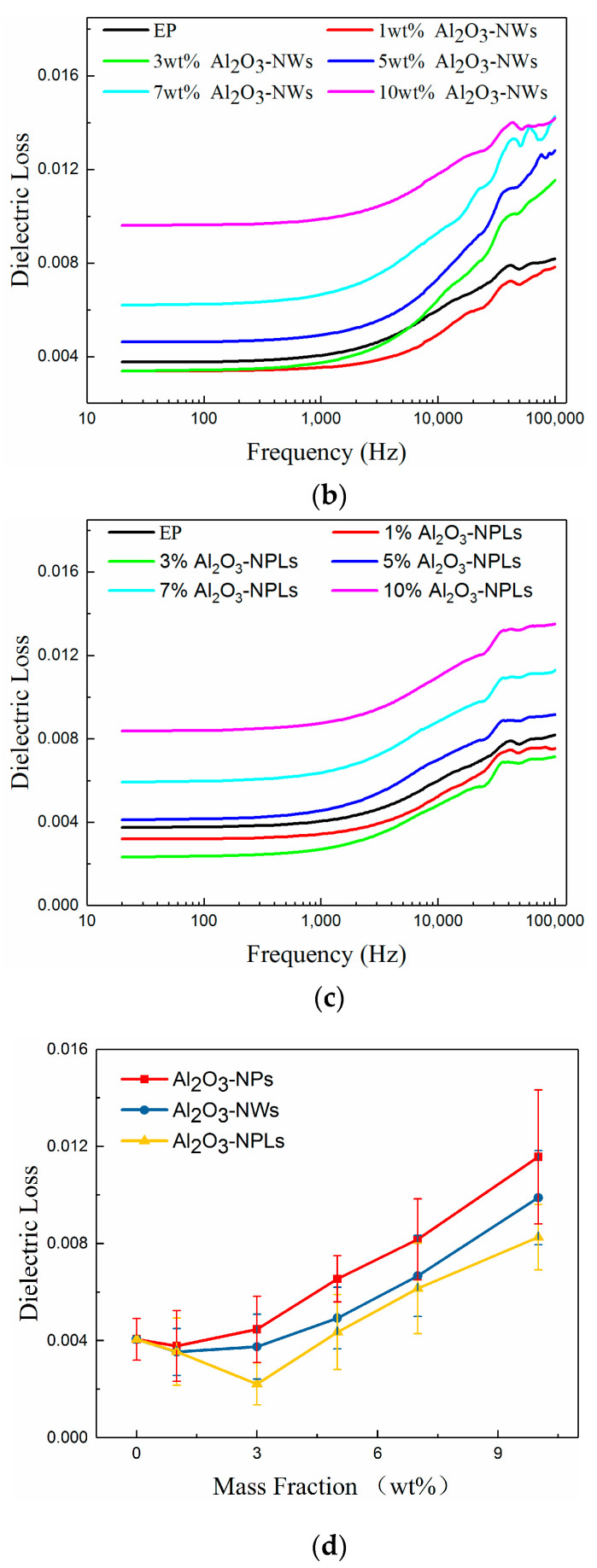
Change in the dielectric losses of the composites with different mass fractions of Al_2_O_3_ nano-fillers with frequency: (**a**) Al_2_O_3_-NPs, (**b**) Al_2_O_3_-NWs, (**c**) Al_2_O_3_-NPLs, and (**d**) dielectric losses of the composites as a function of filler content measured at room temperature and 1 kHz.

In addition to dielectric constant, dielectric loss is an important parameter for studying dielectric properties, since part of the electrical energy is consumed and converted into heat in the applied electric field. The dielectric loss of a composite in an alternating electric field includes conductive and polarization losses, both of which are affected by the interface of the composite. It was concluded that the energy levels and densities of the deep traps introduced by the interfaces of the three composites first increased and then decreased with increasing mass fraction of filler. The mass fraction of the fillers corresponding to the maximum volume resistivity was also different between the three materials due to their different nanostructures. During the polarization process, the mobility of the carriers and the conducting loss of the composite decreased due to the influence of the deep traps introduced at the interface. In other words, the interface hindered the movement of the polar groups and the epoxy resin segments as well as the dipole turning and reduced the polarization loss of the composite. This affected the polarization process, yet the overall dielectric constant increased due to the high dielectric constant of the nano-Al_2_O_3_ filler. However, the nano-filler is prone to agglomeration due to its high specific surface area, and the internal defects of the composites increased with increasing mass fraction of nano-Al_2_O_3_ filler, which weakened the limiting ability in the epoxy resin segment and polar group. Thus, the energy level and density of the deep traps at the interface were reduced, and the dielectric loss of the composite increased. It can be seen from Figure 8 that the dielectric loss of the three samples first decreased and then increased with increasing mass fraction of filler, which is consistent with the above-explained theoretical analyses. It is worth noting that each composite exhibited the minimum dielectric loss at different mass fractions of fillers. More specifically, Al_2_O_3_-NPs/epoxy resin, Al_2_O_3_-NWs/epoxy resin, and Al_2_O_3_-NPLs/epoxy resin reached the minimum dielectric loss at compositions of 1 wt.%, 3 wt.%, and 5 wt.% of filler, respectively. This may be due to the fact that the space between the nano-filler particles with larger particle size was relatively large at constant mass fractions, and the mass fraction required for interfacial overlapping was high. Moreover, fillers with larger particle size were dispersed more uniformly with less agglomeration, so the mass fraction of the filler was linearly proportional with particle size when the three samples reached the minimum dielectric loss, which is consistent with the experimental results.

From the viewpoint of frequency, the relaxation polarization loss caused by the polar groups was significantly observed with changes in frequency, since the epoxy resin matrix contained polar groups, and the addition of Al_2_O_3_ nano-filler introduced new ones, such as -OH. The relaxation polarization exhibited a small lag, and the relaxation polarization loss was insignificant at low frequencies. Additionally, the polarization lag time, the relaxation polarization loss, and the dielectric loss of the composite increased with increasing frequency.

### 3.5. Breakdown Strength

**Figure 9:**
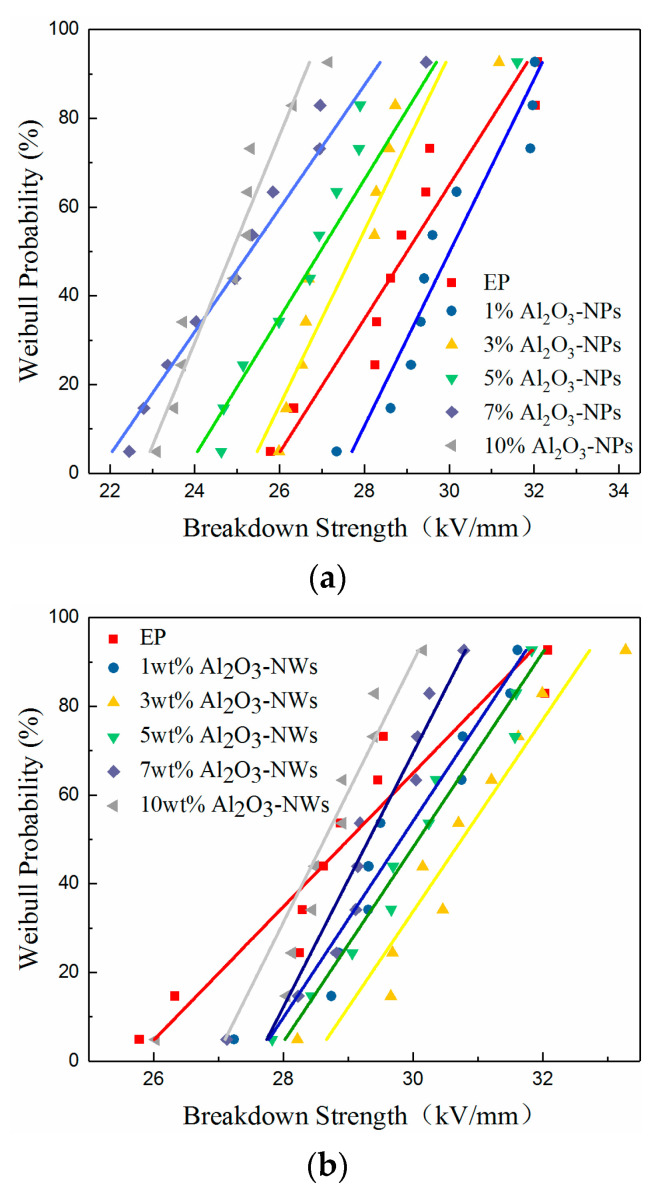

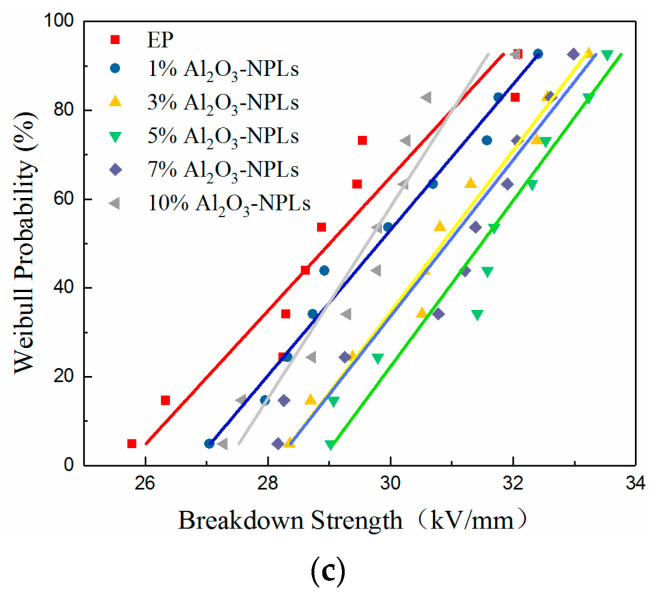
Weibull distribution of composite AC breakdown strength with different mass fractions of Al_2_O_3_nano-fillers: (**a**) Al_2_O_3_-NPs, (**b**) Al_2_O_3_-NWs, and (**c**) Al_2_O_3_-NPLs.

In order to study the influence of Al_2_O_3_ nano-fillers with different nanostructures on the AC breakdown performance of epoxy-based composites, the AC breakdown strength of the three samples was measured, and the data was analyzed and studied by the Weibull distribution. The Weibull distribution was reflected in the breakdown probability of the composites under a certain field strength. For AC voltage breakdown strength, the two-parameter Weibull distribution can be expressed as:

(8) Pf=1−e−(EE0)β

where $P_{f}$ is the cumulative breakdown probability of the sample, *E* is the breakdown field strength, and *E*_0_ is the scale parameter representing the breakdown field strength when the failure probability is 0.632. β is the shape parameter, which is fixed to a positive value, and reflects the distribution range of the breakdown voltage. The discreteness of the breakdown voltage data decreases as β increases. According to the feasibility international standard of IEC/TC56, during the breakdown test, the probability $P_{f}$ can be directly expressed by Equation (9) when the sample size is less than 25.

(9) $$P_{f}=\frac{i-0.5}{n+0.25}$$

where *i* is the *i* th measured breakdown field strength in ascending order and *n* is the number of the measured breakdown field strength [38]. The number of permutations of the breakdown field strength when the breakdown probability equals 0.632 can be obtained by Equation (9), and the corresponding breakdown field strength is the scale parameter *E*_0_ of the Weibull distribution. *E*_0_ is then incorporated into Equation (8), and the shape parameter β of the Weibull distribution can be calculated according to the measured breakdown field strength *E*. The Weibull distribution diagram of the AC breakdown strength of the composite is shown in Figure 9, and the relevant parameters of the Weibull distribution are shown in Table 1.

The AC breakdown of the composites is not a single form of breakdown, but an electrothermal breakdown that is affected by charge movement and heat accumulation. The Al_2_O_3_ nano-filler introduced deep traps in the composites, and the volume resistivity of the three samples first increased and then decreased with increasing mass fraction of filler. The corresponding mass fractions were 1, 3, and 5% for Al_2_O_3_-NPs, Al_2_O_3_-NWs, and Al_2_O_3_-NPLs, respectively, at the maximum volume resistivity. It can also be seen from Figure 9 that the AC breakdown strength of the three samples first increased and then decreased, and the mass fraction of each sample at the maximum breakdown strength was also consistent with the volume resistivity data. This may be due to the fact that the limitation of deep traps on carriers can also hinder the directional movement of carriers and improve the AC breakdown strength of the composite.

On the other hand, AC breakdown is different from DC breakdown. Thermal breakdown is relevant in the process of AC breakdown. The accumulation of heat inside the composite affects the AC breakdown strength of the composite. When the composite material is broken under the action of an AC electric field, the power loss per unit volume p can be expressed as follows:

(10) $$p=E^{2}\omega \varepsilon_{0}\varepsilon_{r}\tan\delta_{0}e^{\alpha (t_{m}-t_{0})}$$

where $\varepsilon_{r}$ is the dielectric constant of the material, $\tan\delta_{0}$ is the tangent value of the dielectric loss angle at 0 °C, α is the temperature coefficient of the dielectric loss angle, *t_m_* is the highest temperature in the medium, and *t_0_* is the temperature of the surrounding environment. Most of the energy loss in the medium is the loss of heat energy. In other words, the thermal energy *Q* produced by the medium can be described as follows:

(11) $$Q=E^{2}h\omega \varepsilon_{0}\varepsilon_{r}\tan\delta_{0}e^{\alpha (t_{m}-t_{0})}$$

It can be seen from Equation (11) that the heat generated inside the composite increases as the dielectric constant and dielectric loss of the composite increase. Since the dielectric losses of the three samples first decreased and then increased, the heat accumulation inside the three samples also reached a minimum near the corresponding mass fraction of minimum dielectric loss, when the effect of thermal breakdown on AC breakdown is minimal. The AC breakdown strength of the three samples first increased and then decreased with increasing mass fraction of fillers, which is consistent with the measured results.

It is worth noting that the maximum breakdown strength of the composite materials decreased in the order of Al_2_O_3_-NPLs > Al_2_O_3_-NWs > Al_2_O_3_-NPs. This is because when nanostructured Al_2_O_3_-fillers are uniformly dispersed in the epoxy resin matrix, most of the Al_2_O_3_-NPLs show the same orientation, and easily form a parallel layered structure, showing the most enhancement on the AC breakdown strength. For Al_2_O_3_-NWs and Al_2_O_3_-NPs, the current forms a path between the end of the nanowires and nanoparticles, and the effect of improving the AC breakdown strength is relatively lower. However, the AC breakdown strength of the composite can still be increased at an appropriate mass fraction.

## 4. Conclusions

In this paper, three kinds of Al_2_O_3_/epoxy resin composites with different nanostructured fillers were prepared and investigated. The effects of the three nano-fillers on the thermal conductivity and insulating properties of the composites were analyzed, and the effect of each nanostructured filler on the related properties was studied. The conclusions are as follows:

1. Nano-Al_2_O_3_ can improve the thermal conductivity of composites. The thermal conductivity of the composites increased with increasing mass fraction of filler, which was higher than that of the pure epoxy resin. Additionally, the thermal conductivity of the composites increased with increasing filler particle size. Moreover, Al_2_O_3_-NWs more easily form parallel heat-conducting paths due to their structure and disordered orientation, which significantly improved the thermal conductivity to a 34% higher value than pure epoxy.
2. A proper amount of nano-Al_2_O_3_ filler can increase the volume resistivity of the composite. The volume resistivity of the composites first increased and then decreased with increasing mass fraction of fillers, but each nanofiller exhibited a maximum volume resistivity for a distinct mass fraction. More specifically, the mass fraction of Al_2_O_3_-NPLs was significantly higher than that of the other two fillers. In addition, Al_2_O_3_-NPLs improved the volume resistivity more significantly than the other fillers, which was 53% higher than pure epoxy resin.
3. The dielectric properties of the composites were enhanced with the addition of fillers. The dielectric constants of the composites increased with increasing mass fraction of fillers, since Al_2_O_3_ has a higher dielectric constant than the epoxy resin. However, the dielectric losses of the composites first decreased and then increased due to the deep traps introduced by the fillers. It was found that the minimum dielectric loss for the Al_2_O_3_-NPLs/epoxy composite was lower than for the other two composites.
4. A proper amount of nano-Al_2_O_3_ filler can improve the AC breakdown performance of the composite. The breakdown strength of the composites first increased and then decreased with increasing mass fraction of filler. Al_2_O_3_-NPLs exhibited the most significant enhancement of the AC breakdown performance of the composites. This may be due to the fact that the volume resistivity and dielectric loss of the composite play a vital role in the electrothermal breakdown process of the composite. The layered structure formed by the Al_2_O_3_-NPLs in the epoxy resin matrix also improved the breakdown strength of the composite.

## Figures and Tables

**Table 1:** Weibull distribution parameters of composite AC breakdown strength with different mass fractions of Al_2_O_3_-NPs, Al_2_O_3_-NWs and Al_2_O_3_-NPLs.

	Al_2_O_3_-NPs		Al_2_O_3_-NWs		Al_2_O_3_-NPLs	
Mass Fraction	*E*_0_	*β*	*E*_0_	*β*	*E*_0_	*β*
0 wt.%	29.5	22.5	29.5	22.5	29.5	22.5
1 wt.%	30.2	30.5	30.7	24.7	30.7	23.7
3 wt.%	28.3	35.3	31.5	29.7	31.3	303
5 wt.%	27.3	28.5	30.3	34.5	32.3	28.0
7 wt.%	25.8	21.3	30.0	29.3	31.9	24.0
10 wt.%	25.3	33.8	28.9	28.5	30.2	29.2
